# Clinical Periodontal Evaluation and Assessment of Dipeptidyl-Peptidase-4 and Galectin-3 in Gingival Crevicular Fluid of Periodontitis Patients with Heart Failure and Diabetes

**DOI:** 10.3390/jcm14103345

**Published:** 2025-05-12

**Authors:** Ana Păvălan, Mihail Virgil Boldeanu, Flavia Mirela Nicolae, Theodora Claudia Gheonea, Ion Rogoveanu, Cristina Florescu, Adina Turcu-Știolică, Dorin Nicolae Gheorghe, Dora Maria Popescu, Andrada Șoancă, Alexandra Roman, Petra Șurlin

**Affiliations:** 1 Department of Periodontology, Research Center of Periodontal-Systemic Interactions, Faculty of Dental Medicine, University of Medicine and Pharmacy of Craiova, 200349 Craiova, Romania; 2 Department of Immunology, Faculty of Medicine, University of Medicine and Pharmacy of Craiova, 200349 Craiova, Romania; 3 Center for IBD Patients, Faculty of Medicine, University of Medicine and Pharmacy of Craiova, 200345 Craiova, Romania; 4 Department of Gastroenterology, Faculty of Medicine, University of Medicine and Pharmacy of Craiova, 200349 Craiova, Romania; 5 Department of Internal Medicine and Cardiology, Faculty of Medicine, University of Medicine and Pharmacy of Craiova, 200349 Craiova, Romania; 6 Department of Pharmacoeconomics, University of Medicine and Pharmacy of Craiova, 200349 Craiova, Romania; 7 Department of Periodontology, Faculty of Dental Medicine, Iuliu Hațieganu University of Medicine and Pharmacy Cluj-Napoca, Victor Babeș St., No. 15, 400012 Cluj-Napoca, Romania

**Keywords:** periodontitis, diabetes mellitus, heart failure, dpp-4, gal-3

## Abstract

**Background/Objectives**: Periodontitis, a prevalent chronic inflammatory disease affecting tooth-supporting structures, has been increasingly linked to systemic conditions such as diabetes mellitus (DM) and cardiovascular diseases (CVDs). This study aimed to evaluate the periodontal status and levels of dipeptidyl peptidase-4 (DPP-4) and galectin-3 (Gal-3) in gingival crevicular fluid (GCF) of patients with periodontitis, heart failure (HF), and diabetes, exploring their potential as biomarkers for disease association. **Methods:** A cross-sectional study was conducted on 88 patients categorized into four groups: periodontally and systemically healthy (control, C); periodontitis (P); periodontitis and HF (P+HF); and periodontitis, HF, and diabetes (P+HF+D). Periodontal status was assessed using probing pocket depth (PPD) and Gingival Index (GI). GCF samples were collected and analyzed for DPP-4 and Gal-3 levels using ELISA. Statistical analyses were performed to assess differences between groups and potential correlations. **Results:** Results indicated significantly higher levels of DPP-4 in all test groups compared to controls (*p* < 0.0001), with the highest levels in the P+HF+D group. Similarly, Gal-3 levels were elevated in periodontitis patients, particularly in those with HF (*p* < 0.0001), and there was no significant difference between P+HF and P+HF+D groups. No significant differences were observed between smokers and non-smokers regarding these markers. Positive correlations were found between the periodontal parameters and the immunological markers in all test groups. **Conclusions:** The findings suggest that DPP-4 and Gal-3 may serve as potential biomarkers for periodontitis in association with heart failure and diabetes, with DPP-4 being more upregulated in the association with diabetes and Gal-3 with heart failure.

## 1. Introduction

As the sixth most frequent condition worldwide, periodontitis is also the most common chronic inflammatory non-communicable disease affecting individuals [1,2]. The tooth-supporting structures gradually deteriorate as a result of periodontal diseases. Its main characteristics include gingival bleeding, periodontal pocketing, radiographically measured alveolar bone loss, and loss of periodontal tissue support, which manifests as clinical attachment loss (CAL) [3]. Although preventable and curable in most situations, it can result in tooth loss if left untreated [2]. The organized aggregation of bacteria living inside a complex intercellular matrix, known as the tooth plaque biofilm, is the principal etiological factor responsible for the onset and development of periodontal disease, with multiple other local and systemic favorizing factors, like smoking and diabetes [2,3,4]. Multispecies biofilms do, in fact, show intricate interactions between the host and the bacteria. Furthermore, not every resident microorganism in the biofilm is harmful; there are beneficial bacteria that work to preserve a symbiotic connection between the host’s immune system and the plaque microbiome. This keeps pathogenic microorganisms from emerging and dysbiosis from developing [4]. The onset and course of periodontal disease have been strongly linked to *Porphyromonas gingivalis*, one of the main pathogens associated with the pathophysiology of periodontal disease, and it has been proposed that its proteolytic enzymes play a role in processes such as invasion, tissue damage, and evasion of host antibacterial responses [5]. The bacterium is capable of creating a variety of dipeptide-producing exopeptidases, dipeptidyl peptidases (DPPs) [6,7,8]. DPP-4 activity was positively correlated with *P. gingivalis* prevalence and clinical periodontal parameters [8]. Previous studies have shown that when clinical indicators rose, DPP-4 assessments increased in those with gingivitis or periodontitis [9,10,11]. Regarding its systemic outcomes, through the breakdown of incretins, bacterial DPP-4 appears to be involved in blood glucose control [5]. Additionally, DPP-4 inhibitors are a class of oral antidiabetic medications that block the breakdown of incretin and improve glucose tolerance and insulin production. These medications are now used to treat type 2 diabetes [12,13].

While a strong causal link between systemic diseases and periodontitis has not yet been established, research suggests that periodontal pathogens and the immuno-inflammatory responses that follow are independently linked to the pathogenesis of a variety of systemic diseases leading to premature death [4,14], such as diabetes mellitus [15], cardiovascular diseases [14], Alzheimer’s disease, chronic kidney disease, rheumatoid arthritis, chronic obstructive pulmonary diseases, certain cancers, and adverse pregnancy outcomes [4,14]. Certain non-communicable diseases, such as diabetes and cardiovascular disease, are now independently linked to severe periodontitis according to a significant amount of research [16,17].

Diabetes mellitus is becoming a worldwide epidemic, with consequences that have a substantial influence on lifespan, quality of life, and healthcare expenses [18,19]. Inflammation precedes the onset of diabetes by causing pancreatic beta-cell malfunction and apoptosis. It also affects the development of insulin resistance and leads to diabetes in the end [19,20,21]. Thus, it makes scientific sense that persistent inflammation that fails to subside caused by periodontal disease affects diabetes management (higher HbA1C) and its complications, like insulin resistance, beta-cell function, and the onset of type 2 diabetes. Furthermore, data suggest that periodontitis exacerbates complications and has a negative impact on diabetes glycemic management [16,22,23]. The deterioration of their clinical status is a concern for patients hospitalized with diabetes mellitus (DM) and heart failure (HF) [24].

Cardiovascular disease (CVD) is currently the world’s leading cause of death and morbidity among noncommunicable illnesses. Currently, around one-third of deaths worldwide are related to CVD, claiming 17.9 million lives annually, according to estimates [13]. The primary lifestyle variables that have historically been associated with cardiovascular disease are still tobacco smoking, dyslipidemia, hypertension, and altered glucose metabolism [13]. The latter are associated with obesity and type 2 diabetes mellitus, two of the main risk factors for myocardial infarction that may be attributed to diets heavy in saturated fats, salt, and refined carbohydrates [13,14,25,26]. It was shown that the scientific plausibility of the relationship between periodontitis and CVD was supported by translocated circulating oral microbiota, which may either directly or indirectly cause systemic inflammation, which in turn influences the development of atherothrombogenesis [14].

Heart failure (HF) is a multifactorial clinical condition with high mortality and morbidity, arising from any component of ventricular filling or blood ejection dysfunction, either structural or functional [27]. Heart failure risk stratification has benefited greatly from the use of the New York Heart Association (NYHA) classification (I—There are no physical activity restrictions; shortness of breath, palpitations, or excessive weariness are not symptoms of regular physical activity. II—Moderate restriction on exercise; comfortable when at rest; and weariness, palpitations, dyspnea, or chest discomfort are the outcomes of regular activity. III—Noted physical activity restriction; cozy while not in use; and excessive physical exertion might lead to symptoms such as exhaustion, palpitations, dyspnea, or chest discomfort. IV—Heart failure symptoms when at rest; and any physical exertion exacerbates the pain) [28,29,30,31]. Using data from the National Health and Nutrition Examination Survey, Wood and Johnson performed a cross-sectional analysis and discovered that self-reported periodontal disease was an independent risk factor for the presence of HF [29,32].

Galectin-3 (Gal-3), an acute phase protein, is a 30 kDa molecule with a single carbohydrate recognition domain and an amino-terminal polypeptide tail region [33,34]. Gal-3 plays an important role in the progression of periodontal disease by upregulating the production of proinflammatory mediators [35] and current research indicates that assessing gingival crevicular fluid (GCF) Gal-3 levels could be helpful in periodontal disease diagnosis [36,37].

Moreover, as a proinflammatory protein that induces beta-cell dysfunction and insulin resistance through its role as a receptor for advanced glycation end products, Gal-3 is important in the transition from a prediabetic to a diabetic state [19,38]. Gal-3 might serve as a predictor of poor prognosis in T1D or T2D diabetic individuals with cardiomyopathy, nephropathy, retinopathy, coronary atherosclerosis, and other complications of diabetes, according to a recent analysis [19].

Gal-3′s essential contribution to the onset and advancement of cardiovascular disorders, such as atherosclerosis, hypertension, myocarditis, and ischemic heart disease, is now well known [30]. Gal-3, which increases endothelial dysfunction and is produced systemically from fibroblasts and macrophages during active inflammation, is a marker of HF, coronary heart disease (CHD), and cardiovascular fibrosis [35,39]. Gal-3 stimulates the migration of monocytes and macrophages, functioning as a chemokine and contributing significantly to the phagocytosis of bacteria. It was proposed that, in comparison to individuals without CHD, those with periodontitis and those with CHD had higher levels of Gal-3, which might serve as a prognostic marker for both conditions [35,39].

The aim of this study was to evaluate the periodontal status and levels of DPP-4 and Gal-3 in the GCF of patients with periodontitis, heart failure, and diabetes, exploring the possibility of using them as biomarkers of these diseases’ associations.

## 2. Materials and Methods

### 2.1. Study Design and Setting

The present research is a cross-sectional, transversal study. It was conducted between March and December 2023. This study was carried out within the University of Medicine and Pharmacy of Craiova, Romania, in the departments of Periodontology, Gastroenterology, Internal Medicine, and Cardiology, in accordance with the STROBE guidelines [40] for study design, after receiving approval from the University Ethics and Scientific Deontology Committee of the University of Medicine and Pharmacy of Craiova and “Filantropia” Clinical Municipal Hospital of Craiova, Romania.

The current study was carried out in compliance with the principles of the Declaration of Helsinki 1975–2013 and adheres to the principles of the General Data Protection Regulation (GDPR) of the European Union regarding the protection of patient data contained in the study groups. An informed consent form was signed by every patient who was included in this investigation.

### 2.2. Participants

This study included 88 patients, who attended the University of Medicine and Pharmacy of Craiova, Romania, within the Periodontology, Gastroenterology, or Internal Medicine and Cardiology Department, where they were diagnosed with periodontitis [3,41], heart failure using NYHA classification [28], or type 2 diabetes [42]. The inclusion criteria were patients with age ≥18 years, regardless of the gender or smoking status, diagnosed with periodontitis stage II-III, heart failure class II-III, type 2 diabetes, or periodontally and systemically healthy. Patients with other diseases or who have taken antibiotics or NSAIDs at least one month before starting the study were excluded. They were split into four groups, three test groups and one control: (i) patients who were diagnosed with diabetes, heart failure, and periodontitis (P+HF+D)-25; (ii) patients who had periodontitis and heart failure (P+HF)-21; (iii) patients with periodontitis (P)-21; and patients who were periodontally and systemically healthy—control group (C)—21 subjects.

### 2.3. Periodontal Examination and Diagnosis

The same well-trained dentist (A.P.) performed an oral and periodontal clinical examination on each patient. From the periodontal chart retained for the statistical analysis, the following variables were determined: pocket probing depth (PPD) and Gingival Index (GI). A UNC15 periodontal probe (Hu-Friedy, Chicago, IL, USA) was used for periodontal examination, with the exception of the third molars and any remaining root tips, at six different sites for every tooth (mesio-vestibular, centro-vestibular, disto-vestibular, mesio-lingual, centro-lingual, and disto-lingual), with respect to the immediate full millimeter. Millimeters were used to express PPD. GI was scored from 0 to 3 (0 = normal gingiva; 1 = mild inflammation, as evidenced by slight color change, slight oedema, and no bleeding upon probing; 2 = moderate inflammation, as evidenced by redness, oedema and glazing, and bleeding on probing; 3 = severe inflammation, as evidenced by marked redness and oedema, ulceration, and a tendency toward spontaneous bleeding) [43].

In accordance with the 2018 classification of periodontal diseases and conditions [3,41], patients were diagnosed with periodontitis if they had interdental clinical attachment loss (CAL) at two or more non-adjacent teeth, or vestibular/oral CAL greater than or equal to 3 mm with PPD greater than or equal to 3 mm in two or more teeth. Patients who failed to meet the prior requirements were regarded as periodontally healthy. Smoking status was noted.

### 2.4. Gingival Crevicular Fluid Sampling

Following the clinical assessment of periodontal disease, the gingival crevicular fluid was harvested from the tooth with the deepest periodontal pocket. Samples of gingival crevicular fluid were obtained by the intracrevicular method using sterile paper strips, (Periopaper, Oraflow, Smithtown, NY, USA) via the absorbing technique. The paper strip was introduced into the gingival sulcus until a minor resistance was detected, and then it was maintained in situ for 30 s. Two samples were performed at one-minute intervals, one for each substance. The exclusion of blood contamination was visually confirmed, and after that, each of them was placed in Eppendorf microtubes containing 50 µL of phosphate-buffered saline (PBS) and stored at −20 °C until it was required.

### 2.5. Immunological Analysis of DPP-4 and Gal-3

The immunological analysis of GCF samples was performed in the Immunology Laboratory of the University of Medicine and Pharmacy in Craiova, Romania, using the ELISA test kits dedicated to the detection and quantitative determination of DPP-4 (E-EL-H6147, Elabscience Houston, TX, USA) and Gal-3 (E-EL-H1470, Elabscience, Houston, TX, USA). Readings were taken with an optical analyzer at 450 nm and corrected at 540 nm.

### 2.6. Statistical Analyses

For the statistical analyses, we used R packages (R Core Team 2022, v. 4.1). We performed descriptive statistics; continuous variables were presented as mean ± standard deviation, median (interquartile range), and range (minimum and maximum), whereas discrete variables were presented as frequencies and percentages. We tested for normality of the continuous variables with the Shapiro–Wilk test (the test rejects the hypothesis of normality when the *p*-value is less than or equal to 0.05). Kruskal–Wallis with Holm adjustment method was performed to evaluate the differences between the four group of patients for DPP-4 and Gal-3. The Brunner–Munzel test was used to assess the differences among smokers and no-smokers. The correlation matrix included the densities for variables (demonstrating their normality) and Spearman correlation coefficients. ROC analysis was performed based on binomial logistic regression, and ROC curve was presented based on multipleROC R package. We computed the DeLong test to compare the area under the curve (AUC) of two correlated ROC curves (e.g., from the same subjects) to determine if one biomarker significantly outperforms the other. A post hoc power analysis was performed using G*Power 3.1 based on differences between two independent means and the effect-size Cohen’s d. A *p*-value less than 0.05 was considered statistically significant.

## 3. Results

### 3.1. Characteristics of Patients from the Four Groups

The age, gender, and smoking status of patients in each group are shown in Table 1.

The age of the 88 eligible patients ranged from 38 to 67 years, distributed by gender: 51 were males (57.95%), and 37 were females (42.04%).

Most of the participants were smokers: 54 (61.36%). The smoking habit was present in all groups at different percentages, but ≥64% in all test groups and 38% in C group.

### 3.2. Differences Between the Assessed Parameters

**Figure 1:**
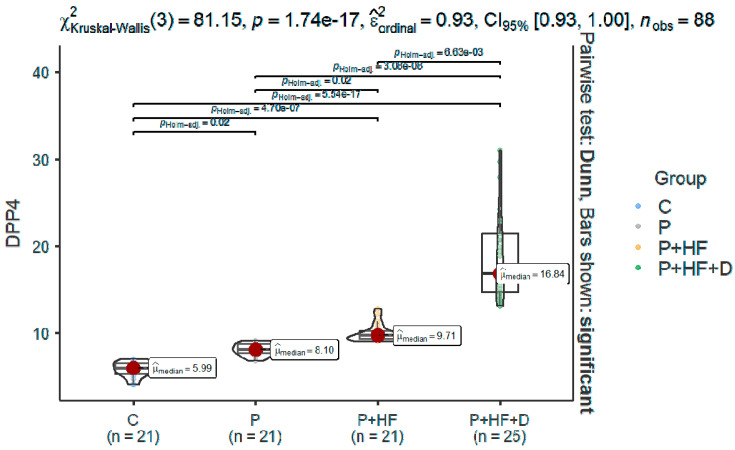
Comparison between groups regarding the DPP4. C, control group; P, periodontitis group; P+HF, periodontitis and heart failure group; P+HF+D, periodontitis, heart failure, and diabetes group; DPP4, dipeptidyl-peptidase-4.

DPP-4 levels were significantly elevated in all test groups compared to the control group (C) (*p* < 0.0001). Specifically, the P+HF group showed 1.22-fold higher DPP-4 levels than the P group (*p* = 0.02). The highest concentrations were observed in the P+HF+D group, which exhibited 1.89-fold and 2.33-fold increases compared to the P+HF (*p* < 0.0001) and P groups (*p* < 0.0001), respectively (Table 2 and Figure 1). The G*Power post hoc analysis test for the two-group independent test (P vs. P+HF) found an achieved power of 0.99, far exceeding the conventional threshold of 0.8. This statistical power indicates near-certainty to detect the observed effect size (d = 2.136) if it exists in the population. The large effect size suggests that the difference in means between groups is substantial and clinically meaningful. In all, 21 samples per group (total *N* = 42) were sufficient to achieve maximal power due to the large effect size. In conclusion, it validates the adequacy of our sample size despite reduced *N*. The same achieved power was calculated for the two-group independent groups P vs. P+HF+D: 1.0, effect size =2.91.

**Figure 2:**
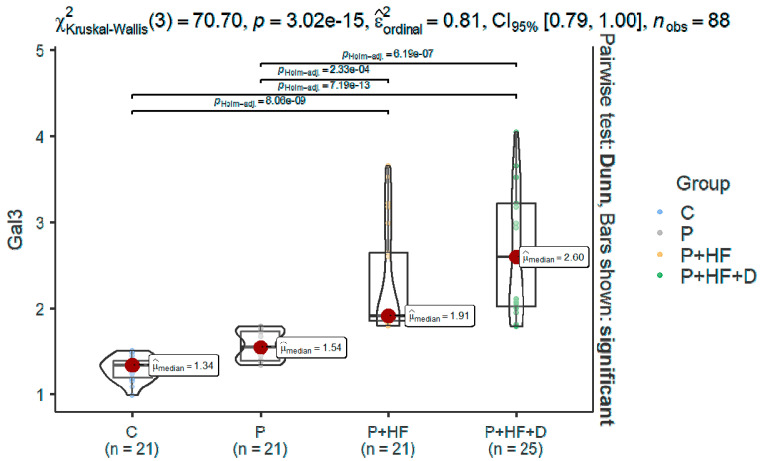
Comparison between groups regarding Gal3. C, control group; P, periodontitis group; P+HF, periodontitis and heart failure group; P+HF+D, periodontitis, heart failure, and diabetes group; Gal3, galectin-3.

Regarding the levels of Gal-3, a significant difference between the four groups was found: the *p*-value between C group and P groups was 0.04, and all the other *p*-values were less than 0.0001 (Table 2, Figure 2), with highest levels in both groups with HF and no significant difference between P+HF and P+HF+D groups. The G*Power post hoc analysis test for two-group independent test (P vs. P+HF) found an achieved power of 0.99, with an effect size of d = 1.94. Additionally, the G*Power post hoc analysis test for the two-group independent test (P vs. P+HF+D) found an achieved power of 0.99, with an effect size of d = 2.03.

In the P, P+HF, and P+HF+D groups, statistically significant differences were not found between the smokers and non-smokers, regarding the assessed parameters, DPP4 (*p* > 0.05) and Gal3 (*p* > 0.05) (Table 3).

### 3.3. Correlations Between the Assessed Parameters

**Figure 3:**
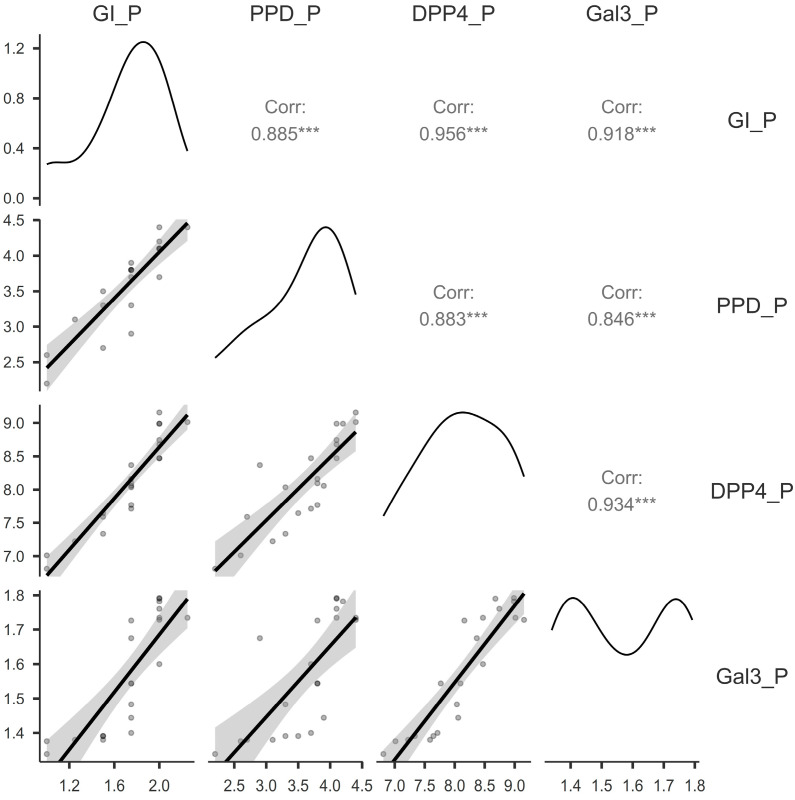
Correlation matrix between the Gingival Index, pocket probing depth, dipeptidyl-peptidase-4, and galectin-3 for the patients with periodontitis. P, periodontitis group; GI, Gingival Index; PPD, pocket probing depth; DPP4, dipeptidyl-peptidase-4; Gal3, galectin-3; ***, *p* < 0.001.

There is a very strong positive correlation between the assessed variables among the patients in the P group, as shown in Figure 3: very strong and statistically significant correlations (*p* < 0.001) were found between GI and PPD (r = 0.88), GI and DPP4 (r = 0.95), GI and Gal3 (r = 0.91), PPD and DPP4 (r = 0.88), PPD and Gal3 (r = 0.84), and DPP4 and Gal3 (r = 0.93).

**Figure 4:**
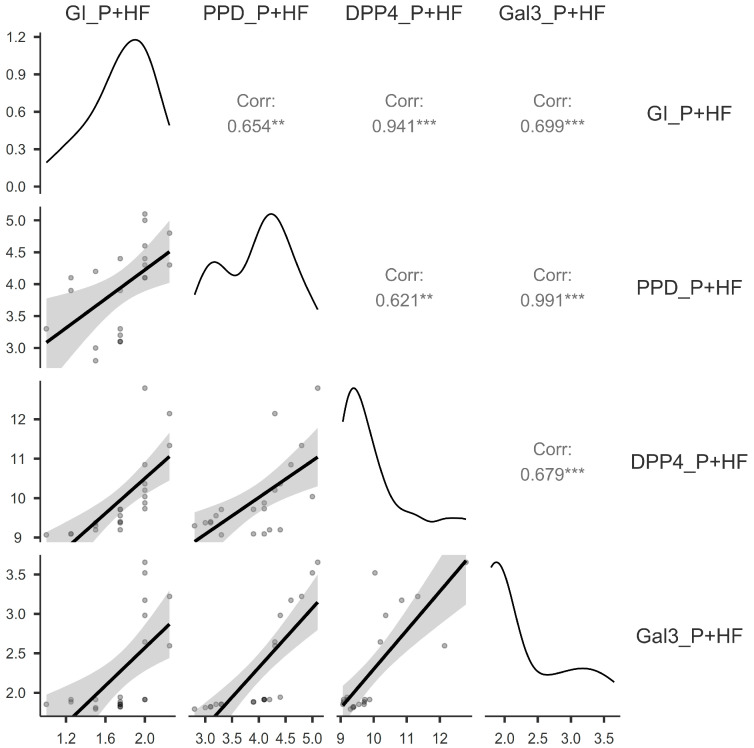
Correlation matrix between the Gingival Index, pocket probing depth, dipeptidyl-peptidase-4, and galectin-3 for the patients with periodontitis and heart failure. P+HF, periodontitis and heart failure group; GI, Gingival Index; PPD, pocket probing depth; DPP4, dipeptidyl-peptidase-4; Gal3, galectin-3; ** *p* < 0.01; *** *p* < 0.001.

There is a positive correlation between the assessed variables in the P+HF group, as shown in Figure 4: very strong statistically significant correlations were found between GI and DPP4 (r = 0.94; *p* < 0.001), and PPD and Gal3 (r = 0.99; *p* < 0.001). Strong statistically significant correlations were found between GI and PPD (r = 0.65; *p* < 0.01), GI and Gal3 (r = 0.69; *p* < 0.001), PPD and DPP4 (r = 0.62; *p* < 0.001), and DPP4 and Gal3 (r = 0.67; *p* < 0.001).

**Figure 5:**
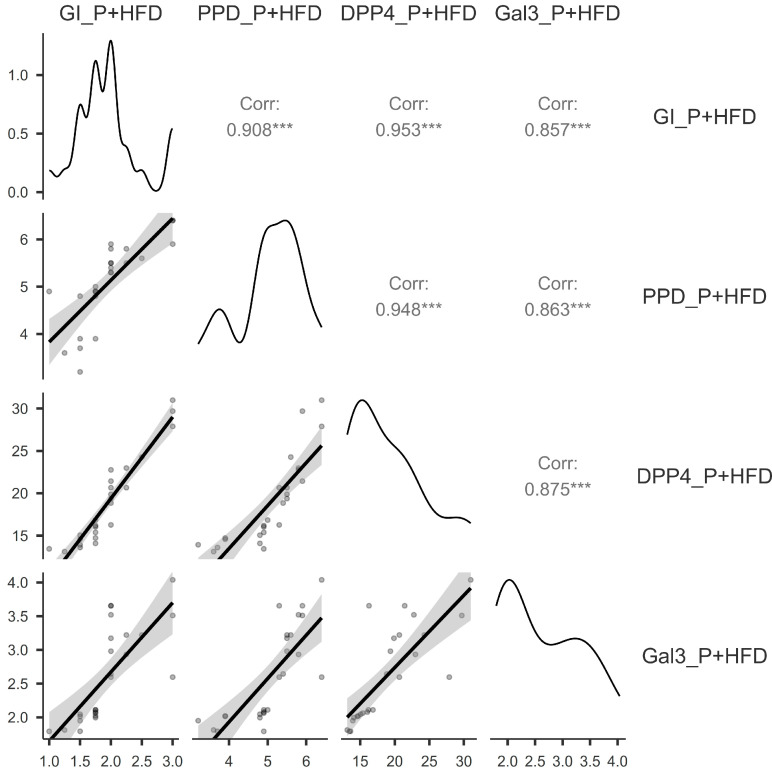
Correlation matrix between the Gingival Index, pocket probing depth, dipeptidyl-peptidase-4, and galectin-3 for the patients with periodontitis, heart failure, and diabetes. P+HFD, periodontitis, heart failure, and diabetes group; GI, Gingival Index; PPD, pocket probing depth; DPP4, dipeptidyl-peptidase-4; Gal3, galectin-3; *** *p* < 0.001.

There is a very strong positive correlation between the assessed variables among the patients in the P+HF+D group, as shown in Figure 5: very strong statistically significant correlations (*p* < 0.001) were found between GI and PPD (r = 0.9), GI and DPP4 (r = 0.95), GI and Gal3 (r = 0.85), PPD and DPP4 (r = 0.94), PPD and Gal3 (r = 0.86), and DPP4 and Gal3 (r = 0.87).

### 3.4. ROC Analysis

DPP-4 showed an exceptional discriminative power for HF in periodontitis patients (AUC, 0.993; 95%CI, [0.978–1]; *p* < 0.001). At a 9.067 ng/mL cutoff, DPP-4 achieves 99% accuracy in distinguishing HF + periodontitis from periodontitis alone.

Gal-3 showed a perfect separation (AUC: 1.0), but it had a non-significant *p*-value (0.183). This indicates potential complete dichotomy in Gal-3 levels between groups and a limited statistical power due to a small HF subgroup size.

**Figure 6:**
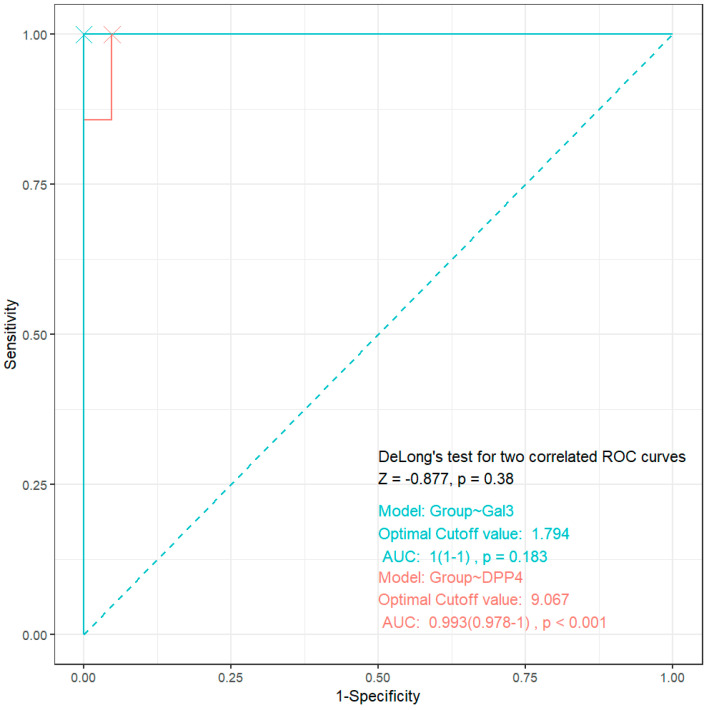
ROC analysis for DPP-4 and Gal-3 as gingival crevicular fluid biomarkers in detecting heart failure in patients with periodontitis.

The DeLong test results (Z = −0.877, *p* = 0.38) showed no significant difference in AUCs between the two biomarkers (Gal-3 and DPP-4). Combining both biomarkers (e.g., multi-panel tests) may improve robustness against confounders. The results presented in Figure 6.

**Figure 7:**
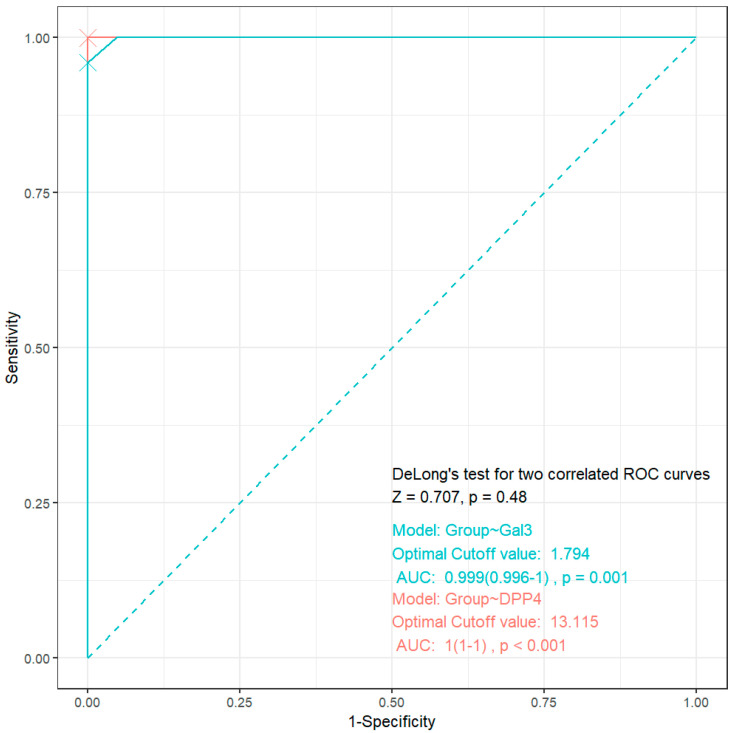
ROC analysis for DPP-4 and Gal-3 as gingival crevicular fluid biomarkers in detecting heart failure and diabetes in patients with periodontitis.

The results presented in Figure 7 suggest DPP-4 as a defining marker for identifying periodontitis patients with associated HF and diabetes (AUC, 1.0; *p* < 0.001) at a 13.115 ng/mL cutoff. Near-perfect accuracy (AUC, 0.999; *p* = 0.001) at a 1.794 ng/mL cutoff supports Gal-3′s utility as a secondary marker.

The DeLong test results (Z = 0.707, *p* = 0.48) hint that DPP-4 may trend toward superiority, but this is not statistically supported in our cohort. Despite DPP-4′s numerically higher AUC (1.0 vs. 0.999), the test confirms no significant difference in diagnostic performance (*p* > 0.05).

## 4. Discussion

In the oral cavity, a majority of bacteria expressing DPP genes concentrate in the subgingival crevice. Anaerobic rods in the human oral microbiota are important residents that generate subgingival dental plaque, and previous data show that *P. gingivalis*-type DPPs, particularly DPP-4, are usually distributed among them [5]. Although we did not search for *Porphyromonas gingivalis* in GCF, as it was not the aim of this study, previous research has shown that the bacterium was found in periodontally healthy patients, but mostly in periodontitis-affected patients [11]. Additionally, it has been demonstrated that *P. gingivalis* DPP-4 increases the activity of host-derived matrix metalloproteinases (MMP)-1 (collagenase) and -2 (gelatinase), which are hypothesized to be implicated in the breakdown of connective tissue in periodontal disease [44]. Periodontal disease-related bone and connective tissue damage could be attributed to human DPP-4 and the DPP-4-like enzyme of *P. gingivalis* [45,46]. Aemaimanan et al. stated that the percentage of sites positive for *P. gingivalis* was correlated with DPP-4 activity [11].

DPP-4 activity in gingival tissue and GCF has been shown in earlier biochemical investigations [47]. One group has localized DPP-4 to fibroblasts, macrophages, and T4 and T8 cells using the same substrate for histochemistry and monoclonal antibodies for immunocytochemistry and immunogold electron microscopy [48]. The enzyme is mostly found on the surfaces of cells and appears to be retained as an active enzyme. It is present in GCF smears surrounding the cell surface in macrophages and T4 and T8 lymphocytes that have moved into the gingival sulcus [6,7,48]. Therefore, GCF DPP-4 may originate from gingival tissue either through the inflammatory exudate or via migrating cells into the crevice [6,7]. In GCF, DPP-4 levels are expected to be greater during disease activity, as both of these sources are expected to increase as the disease progresses, which may lead to its association with the advancement of periodontal disease [48,49]. The activity of GCF DPP-2 and -4 (enzyme per 30 s and enzyme concentration) has been strongly shown by Eley et al. to be predictive of periodontal attachment loss in the future [49].

Aemaimanan revealed a positive association between periodontal clinical measures (probing depth, clinical attachment loss, and bleeding on probing) and the DPP-4 activities [11]. This is consistent with other research which stated that in individuals with gingivitis or periodontitis, DPP-4 rose when clinical parameters increased [9,10]. Elgun et al.’s study found a significant correlation between periodontitis patients’ probing depth and DPP-4 [7]. Probing depth also showed the strongest associations with post-treatment enzyme levels among the other parameters. Because proteolytic enzymes could lead to destruction, they could be considered potential indicators of periodontal disease’s activity [50].

Our results revealed that DPP-4 levels were higher and significantly different in all groups of periodontitis patients compared with those from the control group, with the upregulation of this compound suggesting that its levels are associated with periodontitis’ inflammatory activity. These levels correlate strongly with periodontal parameters in all test groups, pointing to the possibility that DPP-4 activity reflects the degree of periodontal damage and a cumulative effect of the overall low-grade inflammation which is present in all of them.

It has been demonstrated that in individuals with untreated chronic periodontitis, DPP-4-like activities in GCF strongly correlate with clinical parameters of disease severity [5,50]. Both the total protease activity and all clinical parameters decreased after therapy, and a majority were statistically significant at both the patient and site levels [50]. Another study revealed that in the numerous interpatient, intrapatient, and weighted site-level comparisons, positive associations were discovered between DPP-4 and probing and radiographical assessments of periodontal attachment loss in untreated chronic periodontitis patients. When bone and probing attachment were lost, there was a considerable increase in both the GCF volume and overall enzyme activity [51].

The human body’s enzyme dipeptidyl-peptidase-4 is essential for the metabolism of glucose. The vascular system, including endothelial cells, macrophages, cardiomyocytes, smooth muscle cells, valve interstitial cells, and other cell types, expresses DPP-4 widely, suggesting that it may be involved in the development and progression of cardiovascular diseases. Interestingly, a growing body of research has discovered that DPP-4 inhibitors might prevent a range of cardiovascular conditions, including HF, coronary atherosclerosis, calcified aortic valve disease, and hypertension [13].

Most diabetic people commonly suffer from hypertension in addition to their diabetes. The hallmark of hypertension is endothelial dysfunction, and a major factor in the etiology of hypertension is either an increase in endothelium-dependent contraction or a reduction in endothelium-dependent relaxation. In addition to lowering blood glucose, DPP-4 inhibitors are helpful in controlling blood pressure without raising a patient’s heart rate. Clinical investigations and animal models both successfully verified this conclusion [13].

Our study showed that, even though all test groups displayed raised values of DPP-4, the highest levels were in diabetic group, almost two-fold, and then in the periodontitis or periodontitis + heart failure group, confirming the previous findings of the literature presented of its upregulation in the presence of diabetes.

One of the leading causes of mortality globally is HF, and a noteworthy observation is that individuals with HF have a high rate of diabetes. On the other hand, even in the absence of complex coronary artery disease, concomitant diabetes significantly impacts the prognosis for HF [13,52]. Diabetes mellitus affects 28% to 44% of patients with HF, and regardless of ejection fraction, it is associated with negative consequences, such as an increased risk of cardiovascular mortality and hospitalization for HF [53]. Diabetes and HF are closely related conditions. In large-scale clinical studies in individuals with HF, the incidence of diabetes was about 30%. Furthermore, it has been shown that among individuals with type 2 diabetes, the prevalence of HF is greater than that of myocardial infarction or stroke. Diabetes patients often have severe consequences from HF, and HF is a leading cause of mortality for these individuals. In contrast, diabetic problems represent a distinct predictor of death in individuals with HF [12]. Diabetes-related HF is currently referred to as “diabetic cardiomyopathy”. A recent cohort study of the UK’s largest research platform of linked electronic medical records, which included 1.9 million participants, showed that peripheral arterial disease and HF are the most prevalent early signs of cardiovascular disease in people with type 2 diabetes [52].

Antihyperglycemic medications have been used in around 75% of patients with diabetes mellitus and HF. Insulin is the most frequently recommended drug, irrespective of renal function class, and is followed by metformin and sulfonylureas. Additionally, it was demonstrated that thiazolidinediones, as well as certain DPP-4 inhibitors, like saxagliptin, which are antihyperglycemic medications that may exacerbate HF, are administered in one-tenth of HF patients, based on data that became available after the research period, in 2013 [12,53].

While better glucose management is linked to a decreased risk of microvascular difficulties from diabetes mellitus, physicians must consider these possible advantages against the possibility of side effects from antihyperglycemic drugs. For instance, patients with diabetes mellitus who have HF have higher rates of hypoglycemic episodes than patients without HF [53]. The Food and Drug Administration decided to take this into consideration and consider labeling products by manufacturers to disclose this risk [54,55]. Patel et al. showed that while the percentage of patients using antihyperglycemic medications other than insulin (39.5%), sulfonylureas (32.4%), and metformin (17%) was low overall, 6.6% of patients with HF used thiazolidinediones, and 5.1% of patients used dipeptidyl peptidase-4 inhibitors, with trends showing a decrease in thiazolidinedione use and an increase in dipeptidyl peptidase-4 inhibitor use over time [53].

Long-term, safe blood glucose reduction can be achieved by oral antidiabetic medications called dipeptidyl peptidase-4 inhibitors [12,13]. DPP-4 inhibitors are currently the oral antidiabetic medications that doctors most commonly prescribe to patients with type 2 diabetes in Japan. However, the findings remain debatable because some research indicated that they are safe for cardiovascular use, and others suggested the contrary [12,13]. A growing body of research indicates that DPP4 inhibitors may be beneficial in treating a number of cardiovascular conditions, such as HF, coronary atherosclerosis, calcified aortic valve disease, and hypertension. DPP-4 inhibitors, on the one hand, help reduce cardiovascular risk factors and improve blood glucose regulation. Conversely, through a number of pathways, DPP-4 inhibitors directly control the development and course of cardiovascular disorders [13]. The findings of many studies on cardiovascular outcomes have indicated that some DPP-4 inhibitors can raise the chance of being admitted to the hospital due to HF. HF is the most common cardiovascular condition in individuals with diabetes, and it is also a potentially deadly consequence that lowers quality of life [12].

More than half of ambulatory individuals with HF have chronic kidney disease, which may raise the risk of HF with DPP-4 inhibitors like saxagliptin. However, saxagliptin has been examined and appears not to raise the risk of ischemic events in patients with renal insufficiency [53,56].

One of the main conditions endangering human life is coronary atherosclerosis, as is widely recognized. Fortunately, research on animals and in humans has shown that DPP-4 inhibitors can prevent atherosclerosis. In animal models, DPP-4 inhibitors, independent of diabetes, decreased the area of atherosclerotic plaque in ApoE and LDLR knockout mice. DPP-4 inhibitors significantly reduced the development of coronary atherosclerosis in type 2 diabetic patients in clinical trials [13,19,57].

Independent of diabetes, it has been found that DPP-4 inhibitors reduce cardiac fibrosis. Depressing renal and hepatic fibrosis has been found to occur with DPP4 inhibitors. In agreement with these ideas, the DPP family (DPP-8/-9) has both fibroblast activating protein and collagenase properties, and DPP-8/-9, DPP-4 is additionally known to have collagenase capabilities [52]. DPP-4 itself has anti-thrombotic characteristics and may be active on endothelial cells as an immobilized anticoagulant [52]. DPP-4 inhibitors are thought to raise the risk of HF by causing sympathetic activation. Sitagliptin and alogliptin both reduce the renal sodium–hydrogen exchanger 3 function and are mostly eliminated in the urine, in contrast to other DPP-4 inhibitors. Comprehensive cardiovascular outcome studies showed that neither of these two medications raised the likelihood of HF hospitalization [12,13]. Though its precise mechanism is still unknown, research has shown that DPP-4 inhibitors can also control lipid metabolism and lower triglycerides, low-density lipoprotein, and free fatty acid [13].

Bacterial DPPs may be engaged in host physiological activities, in addition to producing dipeptides for bacterial feeding, habitat segregation, and adaptability. The aforementioned discovery is noteworthy in light of the molecular processes behind the associations between periodontal and systemic disorders, which are characterized by the breakdown of physiologically active peptides and proteins by oral bacteria that reach the bloodstream through periodontopathic lesions [5]. It was shown that DPP-4 from *P. gingivalis*, *Tanerella forsythia*, and *Prevotella intermedia* cleaves the N-terminal dipeptide from the incretin peptide hormones GLP-1 and GIP in a manner similar to that of human DPP-4 in a mouse model. The reduced proteolysis of incretins by bacterial DPP-4 resulted in a decline in blood insulin levels and an increase in postprandial hyperglycemia because incretins increase insulin production from pancreatic cells after food intake [5,13]. Furthermore, the incretin action has been enhanced by the development of injectable GLP-1 receptor agonists [12]. These results clearly suggested that periodontopathic bacterial DPP-4 is also involved in type 2 diabetes patients’ glucose control [5,12]. The AGE/RAGE axis is one of the main mechanisms that promote micro-vasculopathy. Interestingly, a new connection has been found between DPP-4 and the AGE/RAGE axis. Diabetes increases the AGE/RAGE axis-related DPP-4 activity. Recent research constantly indicates that DPP-4 inhibitors’ anti-inflammatory properties help to mitigate macro- and micro-vascular endothelial damage in diabetes [52].

Current research indicates that DPP-4 inhibitors can help with hyperlipidemia, hypertension, calcified aortic valve disease, and coronary atherosclerosis; however, their potential benefit for HF is still up for debate. Previous research indicates that DPP-4 inhibitors regulate endothelial function, inflammation, oxidative stress, and vasodilatation through both GLP-1-dependent and -independent mechanisms, hence exerting cardioprotective benefits [13].

Our results suggest that a DPP-4 cutoff of 9.067 ng/mL could guide referral protocols for cardiac evaluation in periodontitis patients. DPP-4′s dual role in glucose metabolism (diabetes) and cardiac pathology (HF) may explain its diagnostic supremacy for identifying periodontitis patients with HF + diabetes association at a 13.115 ng/mL cutoff. All human tissues; fibroblasts; osteoblasts; osteoclasts; endothelium cells; epithelial cells; and immune cells, such as mast cells, dendritic cells, activated T and B cells, macrophages, and monocytes express Gal-3 [58,59]. Numerous biological processes, including immune cell homeostasis, cell proliferation, fibrosis, inflammation, apoptosis, angiogenesis, pre-messenger ribonucleic acid addition, differentiation, and host defense, are dependent upon Gal-3 [19,34,35,60]. Gal-3 can alter the composition of the microbial population in the oral cavity by directly attaching to microorganisms and modifying their clearance. In the gingiva and gingival sulcus, Gal-3 also modifies the activity of many immune cells, which may have an impact on immunological homeostasis [59].

Gal-3 is an essential aspect of immune cell homeostasis and functions as a modulator of cell signaling and cell surface functioning in a range of inflammatory conditions [34,59,61]. By stimulating the production of proinflammatory mediators throughout the periodontal inflammatory process, the proinflammatory protein Gal-3 may contribute to the advancement of inflammation [37,62]. Activation, chemotaxis, and cytokine release of inflammatory cells have all been enhanced by this effective pro-inflammatory signaling molecule [19,59]. Previous research has found an increase in Gal-3 levels in the GCF of periodontitis patients [37,61,63].

Recently, one study showed that Gal-3 and IL-1β were detected in periodontally healthy, gingivitis, and periodontitis participants. When compared to gingivitis and periodontally healthy controls, groups with periodontitis exhibited significantly higher total GCF Gal-3 levels. According to the investigators, the pathophysiology of periodontal diseases is influenced by GCF Gal-3 levels. In differentiating Grade B from C of Stage 3 periodontitis from gingivitis and periodontal health, Gal-3 demonstrated remarkable diagnostic performance [36].

The findings of a recent investigation on the levels of Gal-1 and Gal-3 GCF together strengthen and elaborate upon the hypothesis regarding their vital role in the advancement of periodontal disease. Salivary Gal-3 levels were substantially higher in periodontitis patients than in healthy individuals [37,39]. They may also function as diagnostic markers for periodontal disease, which could be used clinically as a chairside diagnostic tool due to their exceptional diagnostic accuracy for the identification of periodontal diseases and their acceptable ability to measure periodontal disease activity and severity of inflammatory status. Furthermore, they can be used for monitoring the efficacy of treatment since periodontal therapy substantially decreases their levels [62]. As it is essential for immune cell homeostasis and regulates cell communication and cell surface functioning in a variety of inflammatory situations, GCF Gal-3 was shown to be elevated in individuals with periodontitis, according to a recent study by Hendek et al. [63].

In comparison to periodontal healthy individuals, there were statistically significant increased levels of Gal-3 in the GCF and saliva of periodontitis patients. Furthermore, the group with gingivitis exhibited elevated levels in comparison to the healthy group; nevertheless, no statistically significant difference was found between gingivitis and periodontitis. According to the scientists’ findings, Gal-3 is a proinflammatory protein that could prove essential in the development of periodontal disease [37].

Furthermore, the increased Gal-3 levels in periodontitis may also be connected to the association between Gal-3 and *P. gingivalis*. According to Miyauchi et al., *P. gingivalis* lipopolysaccharide stimulated the expression of Gal-3 in placental cells; as a result, *P. gingivalis* in periodontitis lesions could enhance the production of Gal-3 [37,64].

Gal-3 is an essential component in the development of fibrosis, the atherosclerotic process, and inflammation-based disorders in cardiovascular illnesses [30,31,34]. A key role for Gal-3 has been recognized in both the development and progression of atherosclerosis and HF [30]. Mostly due to its involvement in brain and heart ventricular remodeling, Gal-3 is implicated in the pathogenesis of HF and stroke [31,34].

It has recently been demonstrated that there is a positive correlation between Gal-3 concentration and the incidence of HF [31]. According to the 2017 American Heart Association Guidelines for the Management of HF [65], the Gal-3 serum level has been acknowledged as a diagnostic marker for risk stratification and prognosis evaluation of HF patients [66]. Gal-3 is a useful prognostic indicator for HF with both reduced and preserved ejection fraction in both acute and chronic HF [31]. On average, HF patients at low and high risk for clinical consequences may be distinguished using the Gal-3 serum level threshold of 17.8 ng/mL [66]. Increases in Gal-3 levels over the observation period were linked to all-cause mortality, CVD, and HF in the general population [34,67]. According to Zaborska et al., there is a connection between elevated Gal-3 concentration and an increased risk of complications, as well as cardiovascular and all-cause death [31]. Patients with HF were shown to have higher expression of this chimeric lectin, which is linked to increased inflammation and fibrosis. Gal-3 induces HF via a number of processes, such as collagen formation, fibroblast proliferation, infiltration of inflammatory cells, and hypertrophy of cardiomyocytes [30,31].

We found robust correlations between GCF biomarkers (DPP-4 and Gal-3) and clinical measures (GI and PPD), suggesting that these molecules reflect periodontitis severity and may participate in its pathogenesis. DPP-4′s near-perfect alignment with GI (r = 0.95) positions it as a potential surrogate for gingival inflammation, while Gal-3′s slightly lower correlations hint at broader systemic roles. Future studies should test whether targeting these molecules modulates periodontal outcomes.

HF dramatically intensifies the PPD–Gal-3 relationship (r = 0.99), suggesting cardiac comorbidity amplifies Gal-3′s role in periodontal breakdown.

The uniformly high correlations in P+HF+D patients suggest that diabetes intensifies the inflammation–destruction axis, with DPP-4 serving as the lead biomarker. Its near-perfect alignment with GI (r = 0.95) and PPD (r = 0.94) positions it as a potential cornerstone for comorbidity screening, while Gal-3′s slightly weaker links (r = 0.85–0.86) reflect ancillary inflammatory roles.

Our results showed elevated levels of Gal-3 in the GCF of all groups with periodontitis compared with healthy group, but with highest values in both groups with heart failure, with no difference in relation to diabetes, emphasizing its role in periodontal disease, but especially in their association with heart failure.

According to a recent study, type 2 diabetes may have an impact on the relationship between high Gal-3 levels and cardiovascular events. Gal-3 plasma levels were, in fact, linked to cardiovascular events in individuals with diabetes who had coronary artery disease [68].

A positive association between Gal-3 serum levels and body mass index (BMI) was seen in a cohort of individuals with acute HF who were classified based on their BMI. Nevertheless, Gal-3 did not correlate with other BMI categories and was only significantly associated with 30-day mortality or HF readmission in patients with normal BMI (18.5–24.9 kg/m^2^) [31,69].

Although further clinical research is required to fully understand the potential of Gal-3 inhibitors for CVD prevention, their use as therapeutic agents is an appealing and novel idea [30,31,34].

The results of our study revealed no differences between the levels of both markers, DPP-4 and Gal-3, in all groups, regarding the smoking habit; even smoking is considered one of the most important risk factors for periodontitis [3,70] and CVD [13,71]. A recent analysis of the literature concluded that there are no relevant data suggesting the implication of smoking in the higher risk for diabetes [72]. These findings could imply that the pathways of smoking and expression of DPP-4 and Gal-3 could not interfere, but more detailed studies are needed.

The reduced number of participants in this study, derived from the selection criteria applied for greater accuracy, did not make it possible to subdivide them into subgroups according to the degree of periodontitis or to perform correlations with other parameters with high variability, such as those measuring metabolic control. Considering the present study as a preliminary one, our results suggest that the studied markers could be useful in practice, especially by combining them into a panel test. Further study on a larger sample of participants is necessary for better understand their implications in the association of periodontitis with heart failure and diabetes.

## 5. Conclusions

The findings of our study support the evidence that DPP-4 and Gal-3 could be considered markers for periodontitis associated with heart failure and diabetes, with DPP-4 being more upregulated in association with diabetes, and Gal-3 with heart failure, motivating further research.

## Figures and Tables

**Table 1:** Characteristics of patients from the four groups.

	C (*n* = 21)	P (*n* = 21)	P+HF (*n* = 21)	P+HF+D (*n* = 25)
**Age**				
Mean ± SD	47.7 ± 6.16	50.5 ± 5.93	55.4 ± 5.72	59.4 ± 5.63
Median (IQR)	48 (42–52)	51 (46–55)	55 (50–61)	59 (56–64)
Min–max	38–61	43–62	47–66	49–67
**Gender**				
Male (%)	12 (57%)	12 (57%)	11 (52%)	16 (64%)
Female (%)	9 (43%)	9 (43%)	10 (48%)	9 (36%)
**Smoking,** yes (%)	8 (38%)	15 (71%)	15 (71%)	16 (64%)

C, control group; P, periodontitis group; P+HF, periodontitis and heart failure group; P+HF+D, periodontitis, heart failure, and diabetes group; *n*, number of patients in each group; %, percentage of patients in each group; SD, standard deviation; IQR, interquartile range; min–max, range.

**Table 2:** Values of inflammatory markers.

Parameters (ng/mL)	C (*n* = 21)	P (*n* = 21)	P+HF (*n* = 21)	P+HF+D (*n* = 25)
**DPP4**				
Mean ± SD	5.84 ± 0.86	8.11 ± 0.69	9.97 ± 1.02	18.9 ± 5.19
Median	5.99 (5.3–6.5)	8.1 (7.6–8.7)	9.7 (9.3–10.2)	16.8 (14.7–21.4)
Min–max	4.09–7	6.81–9.16	9.07–12.8	13.1–31.0
Normality	0.19	0.578	<0.001	0.011
**Gal3**				
Mean ± SD	1.3 ± 0.14	1.57 ± 0.17	2.48 ± 0.64	2.62 ± 0.71
Median	1.3 (1.2–1.4)	1.5 (1.4–1.7)	1.9 (1.8–2.6)	2.60 (2.02–3.22)
Min–max	0.99–1.50	1.34–1.79	1.79–3.65	1.79–4.04
Normality	0.682	0.006	<0.001	0.010

C, control group; P, periodontitis group; P+HF, periodontitis and heart failure group; P+HF+D, periodontitis, heart failure, and diabetes group; *n*, number of patients in each group; DPP4, dipeptidyl-peptidase-4; Gal3, galectin-3; SD, standard deviation; min–max, range.

**Table 3:** The differences between smokers and non-smokers regarding DPP4 and Gal3 for the four groups of patients.

Parameters (ng/mL)	Groups	Smoking	Non-Smoking	*p*-Value
**DPP4**	C	5.79 ± 0.96	5.86 ± 0.83	0.870
	P	8.25 ± 0.57	7.77 ± 0.9	0.368
	P+HF	10.2 ± 1.14	9.48 ± 0.37	0.132
	P+HF+D	20.2 ± 5.66	16.6 ± 3.38	0.128
**Gal3**	C	1.28 ± 0.16	1.31 ± 0.13	0.787
	P	1.59 ± 0.16	1.53 ± 0.19	0.518
	P+HF	2.34 ± 0.64	2.14 ± 0.68	0.387
	P+HF+D	2.68 ± 0.66	2.52 ± 0.83	0.128

C, control group; P, periodontitis group; P+HF, periodontitis and heart failure group; P+HF+D, periodontitis, heart failure, and diabetes group; DPP4, dipeptidyl-peptidase-4; Gal3, galectin-3; *p*-value < 0.05 for statistically significance.

## Data Availability

Data used to support the findings of this study are available from the corresponding author upon reasonable request.
